# TRPV1-Mediated Sensing of Sodium and Osmotic Pressure in POMC Neurons in the Arcuate Nucleus of the Hypothalamus

**DOI:** 10.3390/nu14132600

**Published:** 2022-06-23

**Authors:** Boyang Zhang, Kazuomi Kario, Toshihiko Yada, Masanori Nakata

**Affiliations:** 1 Department of Physiology, Faculty of Medicine, Wakayama Medical University, Kimiidera 811-1, Wakayama 641-8509, Japan; zhangby@wakayama-med.ac.jp; 2 Division of Cardiovascular Medicine, Department of Medicine, Jichi Medical University, Yakushiji 3311-1, Shimotsuke 329-0498, Japan; kkario@jichi.ac.jp; 3 Center for Integrative Physiology, Kansai Electric Power Medical Research Institute, 1-5-6 Minatojimaminamimachi, Chuou-ku, Kobe 650-0047, Japan; toshihiko.yada@kepmri.org; 4 Department of Diabetes, Endocrinology and Metabolism, Gifu University Graduate School of Medicine, Yanagido1-1, Gifu 501-1194, Japan

**Keywords:** melanocortin system, AgRP, POMC, sodium sensing, TRPV1, blood pressure

## Abstract

The central melanocortin system conducted by anorexigenic pro-opiomelanocortin (POMC) neurons and orexigenic agouti-related peptide (AgRP) neurons in the arcuate nucleus of the hypothalamus (ARC) not only regulates feeding behavior but also blood pressure. Excessive salt intake raises the Na^+^ concentration ([Na^+^]) in the cerebrospinal fluid (CSF) and worsens hypertension. The blood–brain barrier is immature in the ARC. Therefore, both AgRP and POMC neurons in the ARC have easy access to the electrolytes in the blood and can sense changes in their concentrations. However, the sensitivity of AgRP and POMC neurons to Na^+^ remains unclear. This study aimed to explore how the changes in the extracellular Na^+^ concentration ([Na^+^]) influence these neurons by measuring the cytosolic Ca^2+^ concentration ([Ca^2+^]_i_) in the single neurons isolated from the ARC that were subsequently immunocytochemically identified as AgRP or POMC neurons. Both AgRP and POMC neurons responded to increases in both [Na^+^] and osmolarity in C57BL/6 mice. In contrast, in transient receptor potential vanilloid 1 (TRPV1) knockout (KO) mice, POMC neurons failed to respond to increases in both [Na^+^] and osmolarity, while they responded to high glucose and angiotensin II levels with increases in [Ca^2+^]_i_. Moreover, in KO mice fed a high-salt diet, the expression of POMC was lower than that in wild-type mice. These results demonstrate that changes in [Na^+^] and osmolarity are sensed by the ARC POMC neurons via the TRPV1-dependent mechanism.

## 1. Introduction

Obesity is subject to environmental and genetic factors and is triggered by an imbalance between energy intake and energy expenditure. Research over the past two decades has shown that an impairment of the melanocortin pathway in the hypothalamus contributes to hyperphagia and the onset of obesity. The hypothalamic melanocortin system is conducted by anorexigenic pro-opiomelanocortin (POMC) neurons and orexigenic agouti-related peptide (AgRP)/neuropeptide Y (NPY) neurons in the arcuate nucleus of the hypothalamus (ARC) [1,2,3]. α-Melanocyte stimulating hormone (α-MSH) is released from POMC neurons and acts on the melanocortin 3/4 receptors (MC3/4R) on the second-order neurons in the feeding center of the nucleus, also known as the paraventricular nucleus of the hypothalamus (PVH). On the other hand, AgRP neurons release both AgRP and γ-aminobutyric acid (GABA), which antagonize the activity of POMC neurons [4,5,6]. The balance of AgRP neurons and POMC neurons maintains the homeostasis of feeding behavior and energy metabolism [7,8].

Research on the central melanocortin system has mainly focused on energy homeostasis, while few studies have focused on cardiovascular control. It is clear that the melanocortin system plays an important role in the control of blood pressure. Antagonism of the MC4R reduces obesity-related hypertension and renal sympathetic nerve activity (RSNA) [9]. MC4R is highly expressed in the PVH as well as in the areas that integrate energy homeostasis and regulates renal sympathetic nerve activity. Chronic hyperleptinemia stimulates the tonic firing rate of MC4R-expressing PVH neurons, resulting in hypertension [10]. Moreover, MC4R deletion also reduces the pressor response to salt loading as well as prevents the inflammatory and renal damage associated with obesity [11,12]. The melanocortin system has also been implicated in the pathophysiology of salt-sensitive hypertension.

From previous epidemiological and experimental studies, it is known that excessive salt intake contributes to hypertension and vascular dysfunction [13]. A high-salt diet increases the extracellular sodium concentrations ([Na^+^]) in the cerebrospinal fluid (CSF) and in the plasma [14,15,16]. A rapid increase in [Na^+^] in the CSF activates the sympathetic nerve system and raise the arterial blood pressure in rodents [16]. Excessive salt intake raises [Na^+^] in the CSF and raises blood pressure. However, it is unclear how the increase in [Na^+^] influences the POMC neurons, core neurons in the melanocortin system.

The transient receptor potential (TRP) channels, which comprise six transmembrane cation-selective ion channels, constitute a superfamily that includes TRPA, TRPC, TRPM, TRPML, TRPP, and TRPV. TRP channels are activated by various physiological stimuli, including temperature, mechanical stress, redox signaling, and osmolarity. Among the TRP family, TRPV1 plays a key role in regulating blood pressure [17,18]. The TRPV1-positive peripheral sensory nerves act against salt overload to maintain normal blood pressure [19,20]. In the hypothalamus, TRPV1 is expressed in the POMC neurons of the ARC [21,22]. The present study aimed to clarify whether the POMC and/or AgRP neurons in the ARC directly sense increases in [Na^+^] and, if so, whether TRPV1 is implicated in Na^+^ sensing. The study also tested the involvement of TRPVI in the response of POMC neurons to increased osmolarity, glucose, and angiotensin II. 

## 2. Materials and Methods

### 2.1. Animals

TRPV1-null (KO) mice were donated by the department of ophthalmology of Wakayama Medical University [23]. We used male KO mice and their wild-type littermates. All mice were genotyped by polymerase chain reaction (PCR) amplification of genomic DNA isolated from tail tips. Male C57BL/6 and KO mice were housed in a temperature- (22 ± 2 °C) and humidity-controlled (40–70%) room with a 12 h light/12 h dark cycle (light on 8:00–20:00). Food (Standard animal chow DC-8; CLEA, Osaka, Japan) and water were available ad libitum. All animal procedures were conducted in compliance with protocols approved by Wakayama Medical University Animal Care and Use Committee, and all experiments were carried out in accordance with the approved protocols.

### 2.2. Measurement of Cytosolic Calcium Concentration ([Ca^2+^]_i_)

Measurement of [Ca^2+^]_i_ was performed as previously described [24]. Single neurons were isolated from mice aged 5–6 weeks old. Briefly, brain slices containing the entire ARC were prepared, and the ARC was excised. The dissected tissues were washed with HEPES-buffered Krebs–Ringer bicarbonate buffer (HKRB) containing 5 mM or 2.3 mM glucose. The tissues were incubated in 20 units Papain (Sigma Chemical Co., St. Louis, MO, USA)/0.4 units DNase/5 mM Glucose/HKRB, 0.75 mg/mL BSA, and 1 mM cysteine for 15 min at 36 °C in a shaking water bath. The isolated tissues were washed 10 times with 5 mM Glucose/HKRB, dispersed by pipetting, and centrifuged at 700 rpm for 5 min. The cell suspension of the precipitate was placed on a cover glass and pt in an incubator and was allowed to stand at 35 °C for 30 min~2 h. [Ca^2+^]_i_ before being measured by ratiometric fura-2 fluorescence imaging. Briefly, single neurons on coverslips were incubated with 2 μmol/l fura-2/AM (Dojin chemical, Kumamoto, Japan) for 30 min in a 35 °C incubator. Then, coverslips were mounted in a chamber and superfused with reagents and HKRB at 1 mL/min. The fluorescence images depicting the excitation at 340 and 380 nm were captured, and ratio (F340/F380) images were produced by the Aquacosmos system (Hamamatsu Photonics, Hamamatsu, Japan). When [Ca^2+^]_i_ changed within 10 min after the addition of agents and their amplitudes were at least twice as large as the fluctuations of the baseline, they were considered responses. Only the neurons that responded to glutamate (10^−5^ M) were analyzed.

After [Ca^2+^]_i_ measurement, the cells on the cover glass were soaked overnight in 4% paraformaldehyde (PFA)/0.1 M phosphate buffer. After washing with phosphate-buffered saline, they were reacted with 3% H_2_O_2_ solution for 10 min to inactivate endogenous peroxidase. After washing with PBS, blocking was performed with 2% BSA/2% normal goat serum/0.1% Triton X 100/PBS solution for 30 min. Rabbit anti-AgRP antibody (Cloud Clone Corp. 1:500) or rabbit anti-POMC antibody (1:500) [25] was added and reacted overnight at 4 °C. After washing with PBS, the cells were reacted with biotinylated goat anti-rabbit IgG antibody (Vector Laboratories. 1:500) for 40 min. After washing with PBS, the reaction was carried out with the ABC reagent VECTASTAIN Elite ABC kit peroxidase (HRP) (Vector Laboratories) for 30 min. After washing with PBS, the color was developed by FAST DAB PEROXIDASE SUBSTRATE TABLET SET (Sigma Aldrich). AgRP neurons and POMC neurons were identified by collating the photographs taken at the time of [Ca^2+^]_i_ measurement with the photographs taken after immunostaining. In brief, at the end of [Ca^2+^]_i_ imaging, we took photographs of all cells in the microscopic field subjected to [Ca^2+^]_i_ measurements. Based on these photographs, the cells in which [Ca^2+^]_i_ was recorded were correlated with the corresponding immunocytochemical results (Supplementary Figure S1).

### 2.3. Real-Time RT-PCR Analysis

Real-time PCR was performed as previously described [26]. At 5 weeks old, mice were fed a high-salt diet containing 8.0% NaCl or a low-salt diet containing 0.3% NaCl (Oriental Yeast Co, Tokyo, Japan) for 24 h. Bilateral arcuate nuclei were removed from C57BL/6 mice and TRPV1 KO mice after being fed a high-salt or low-salt diet for 24 h, and total RNA was extracted using TRIzol (Invitrogen, Carlsbad, CA, USA). After treatment with RQ1 DNase (Promega, Madison, WI, USA), first-strand cDNA was synthesized with ReverTra Ace kit (Takara bio Inc., Tokyo, Japan). Using SYBR Premix Ex Taq II, quantification was performed via the ΔΔCT method with Thermal Cycler Dice (Takara bio, Shiga, Japan), and glyceraldehyde 3 phosphate dehydrogenase (GAPDH) was used as a control. Primers were as follows: AgRP, 5′-GGTGCTAGATCCACAGAACCG-3′, and 5′-CCAAGCAGGACTCGTGCAG-3′; POMC, 5′-CATTAGGCTTGGAGCAGGTC-3′, and 5′-TCTTGATGATGGCGTTCTTG-3′; GAPDH, 5′-GGCACAGTCAAGGCTGAGAATG-3′, and 5′-ATGGTGGTGAAGACGCCAGTA-3′.

### 2.4. Statistical Analysis

All results are shown as the mean ± SEM. For the significant difference test, one-way ANOVA was performed, and then the Bonferroni test was performed as a multiple comparison test. *p* < 0.05 was considered significant.

## 3. Results

### 3.1. AgRP and POMC Neurons in ARC Sense Na^+^

**Figure 1:**
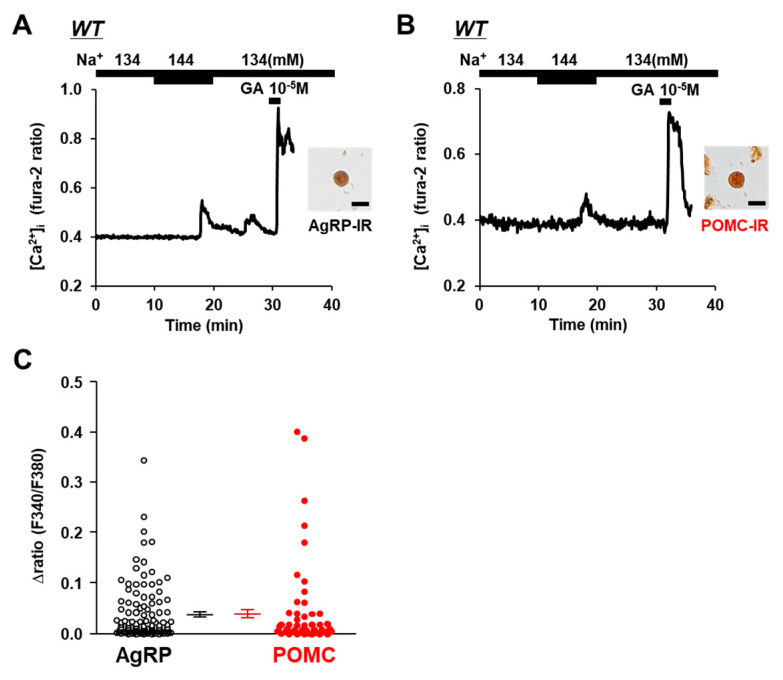
Increased extracellular [Na^+^] increased [Ca^2+^]_i_ in single AgRP and POMC neurons isolated from ARC of wild-type mice.(**A**,**B**) Left panel: Typical examples of [Ca^2+^]_i_ responses to [Na^+^] increase and 10^−5^ M glutamate (GA) in single ARC neurons. Right panel: The single ARC neurons were subsequently shown to be IR to AgRP (**A**) and POMC (**B**). Scale bar is 20 μm. (**C**) Average amplitude of [Ca^2+^]_i_ responses to increased extracellular [Na^+^] (Δratio) in AgRP and POMC neurons. Data are presented as mean ± SEM.

An elevation in the extracellular [Na^+^] from 134 mM to 144 mM increased the amount of [Ca^2+^]_i_ in the neurons that were subsequently determined to be immunoreactive to AgRP (Figure 1A) and immunoreactive to POMC (Figure 1B) in wild-type C57BL/6 mice. Increasing the extracellular [Na^+^] by 10 mM increased [Ca^2+^]_i_, with a Δratio > 0.1 in 13 of 118 (11.0%) of the AgRP neurons and in 7 of 46 (15.2%) of the POMC neurons. The increase in the amplitude of [Ca^2+^]_i_ was not different between the AgRP and POMC neurons (Figure 1C). Thus, both AgRP and POMC neurons directly sense increases in extracellular [Na^+^].

### 3.2. [Na^+^] Sensing in POMC Neurons Is Abolished in TRPV1 KO Mice

**Figure 2:**
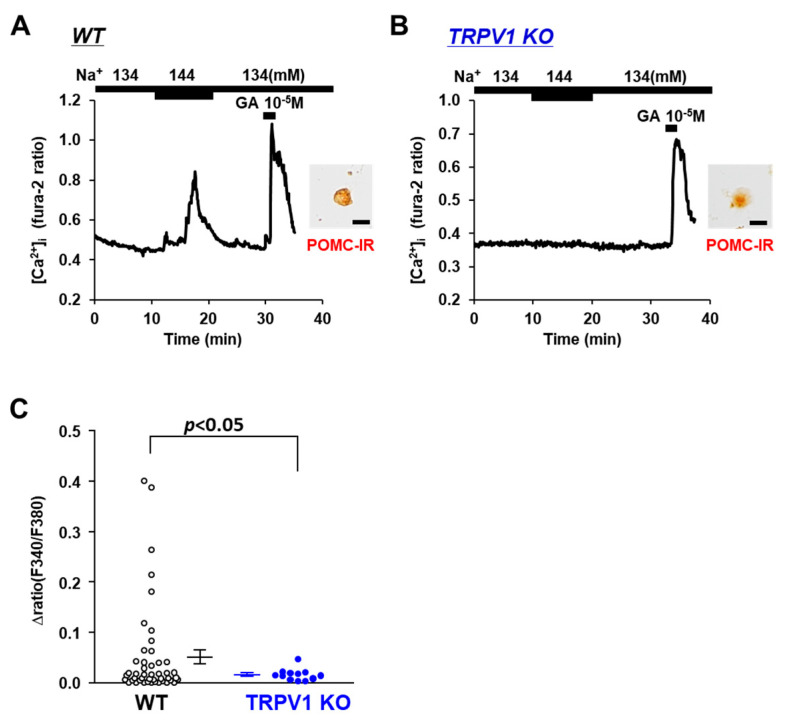
Increased extracellular [Na^+^] failed to substantially increase [Ca^2+^]_i_ in single POMC neurons in ARC from TRPV1 KO mice. (**A**,**B**) Typical examples of [Ca^2+^]_i_ responses to [Na^+^] increase and 10^−5^ M glutamate (GA) in single ARC POMC neuron from wild-type mice (**A**) and TRPV1 KO mice (**B**). Scale bar is 20 μm. (**C**) Average amplitude of [Ca^2+^]_i_ responses to increased extracellular [Na^+^] (Δratio) in POMC neurons. Data are presented as mean ± SEM. WT vs. KO determined by one-way ANOVA followed by the Bonferroni test.

**Figure 3:**
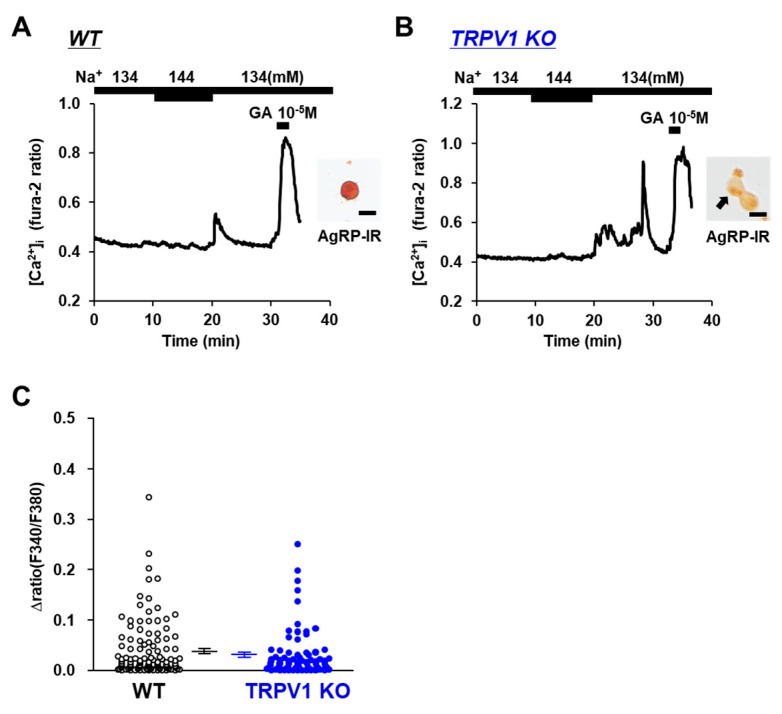
Increased extracellular [Na^+^] increased [Ca^2+^]_i_ in single AgRP neurons from TRPV1 KO mice. (**A**,**B**) Typical example of [Ca^2+^]_i_ responses to [Na^+^] increase and 10^−5^ M glutamate (GA) in AgRP neurons from wild-type mice (**A**) and TRPV1 KO mice (**B**). Scale bar is 20 μm. (**C**) Average amplitude of [Ca^2+^]_i_ responses to increased extracellular [Na^+^] (Δratio) in AgRP neurons. Data are presented as mean ± SEM. WT vs. KO determined by one-way ANOVA followed by the Bonferroni test.

The involvement of the osmotic sensor TRPV1 in extracellular [Na^+^] sensing in POMC and/or AgRP neurons was investigated using TRPV1 KO mice. Elevating the extracellular [Na^+^] from 134 mM to 144 mM increased the [Ca^2+^]_i_ in POMC the neurons of the wild-type mice (Figure 2A), and this response was completely abolished in the POMC neurons isolated from the TRPV1 KO mice (Figure 2B). None of the twelve POMC neurons in the TRPV1 KO mice responded to the 10 mM increase in extracellular [Na^+^] (Figure 2C). Conversely, the elevation of extracellular [Na^+^] from 134 mM to 144 mM increased [Ca^2+^]_i_ in 5 of 78 (6.4%) AgRP neurons in the TRPV1 KO mice and 6 of 118 (5.1%) AgRP neurons in the wild-type mice (Figure 3A,B). No significant differences were observed in the amplitude of the [Ca^2+^]_i_ increase in the AgRP neurons between the C57BL/6 and TRPV1 KO mice (Figure 3C). These results indicated increased TRPV1-dependent sensing of [Na^+^] by POMC neurons but not AgRP neurons.

### 3.3. Osmolarity Increase Activates POMC Neurons via TRPV1

**Figure 4:**
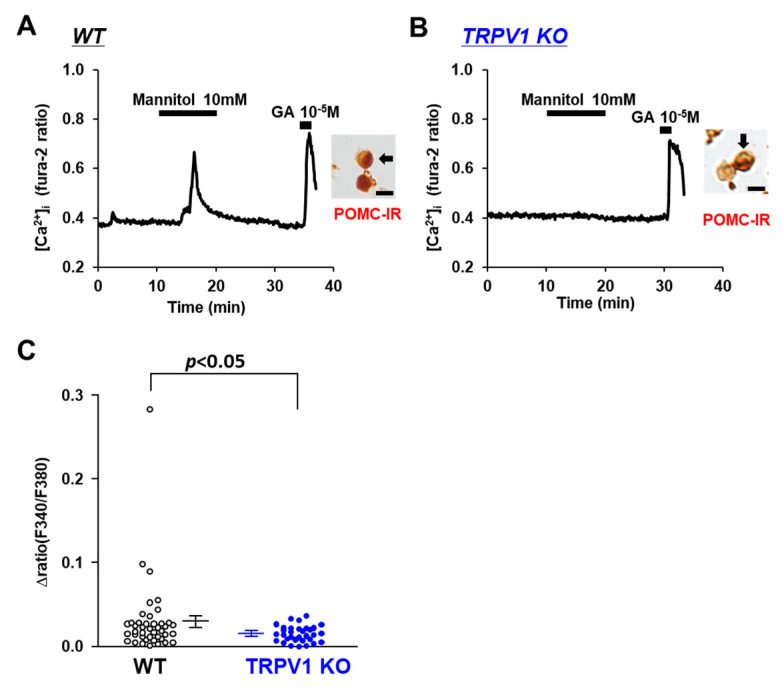
Mannitol increased [Ca^2+^]_i_ in single POMC neurons in ARC. (**A**,**B**) Typical example of [Ca^2+^]_i_ responses to mannitol and 10^−5^ M glutamate (GA) in POMC neurons isolated from wild-type (WT) mice (**A**) and TRPV1 KO mice (**B**). Scale bar is 20 μm. (**C**) Average amplitude of [Ca^2+^]_i_ responses to mannitol (Δratio) in POMC neurons. Data are presented as mean ± SEM. WT vs. KO determined by one-way ANOVA followed by the Bonferroni test.

Na^+^ is the major cation in CSF and the determinant of osmolality. Hence, we examined whether TRPV1 is involved in the sensing of osmotic pressure in POMC neurons. The osmolarity of the extracellular fluid was increased by the addition of mannitol, a sugar alcohol. At 10 mM, mannitol increased [Ca^2+^]_i_ in 5 of 40 POMC neurons from the C57BL/6 mice (Figure 4A), with the amplitude increasing by around 0.05 ratio units, whereas the majority of the POMC neurons in the TRPV1 KO mice did not respond to 10 mM mannitol (Figure 4B). The average amplitude of [Ca^2+^]_i_ increases in the POMC neurons was significantly decreased in the TRPV1 KO mice (Figure 4C). These results show a role of TRPV1 in sensing increases in the extracellular osmolality in POMC neurons.

### 3.4. Glucose-Sensing in POMC Neurons Is Unaltered in TRPV1 KO Mice

**Figure 5:**
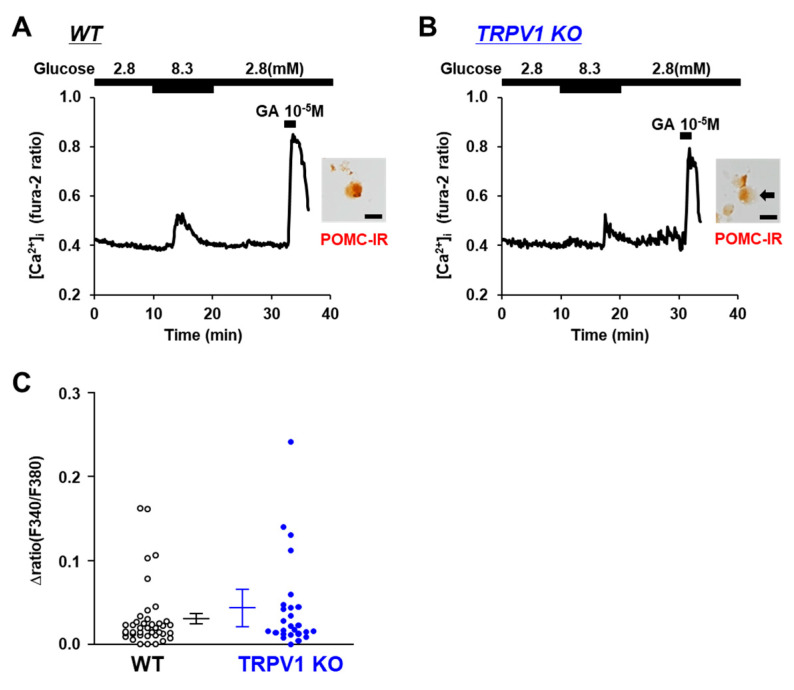
Glucose increased [Ca^2+^]_i_ in single POMC neurons. (**A**,**B**) Glucose (8.3 mM) increased [Ca^2+^]_i_ in POMC neurons from wild-type (WT) mice (**A**) and TRPV1 KO mice (**B**). Scale bar is 20 μm. (**C**) Average amplitude of [Ca^2+^]_i_ responses (Δratio) in POMC neurons. Data are presented as mean ± SEM. WT vs. KO determined by one-way ANOVA followed by the Bonferroni test.

POMC neurons are known to respond to increases in ambient glucose concentrations and are characterized as glucose-excited or glucose-responsive neurons [27,28]. Hence, we examined the response to glucose, a sugar that is metabolized intracellularly. The increase in the glucose concentration from 2.8 mM to 8.3 mM increased the [Ca^2+^]_i_ in the POMC neurons from both the TRPV1 KO and C57BL/6 mice (Figure 5A,B). Furthermore, there was no significant difference in the amplitude of the [Ca^2+^] increases between the C57BL/6 mice and TRPV1 KO mice (Figure 5C). These results indicate that the response to glucose was independent of TRPV1.

### 3.5. Angiotensin II Activates POMC Neurons in WT and TRPV1 KO Mice

**Figure 6:**
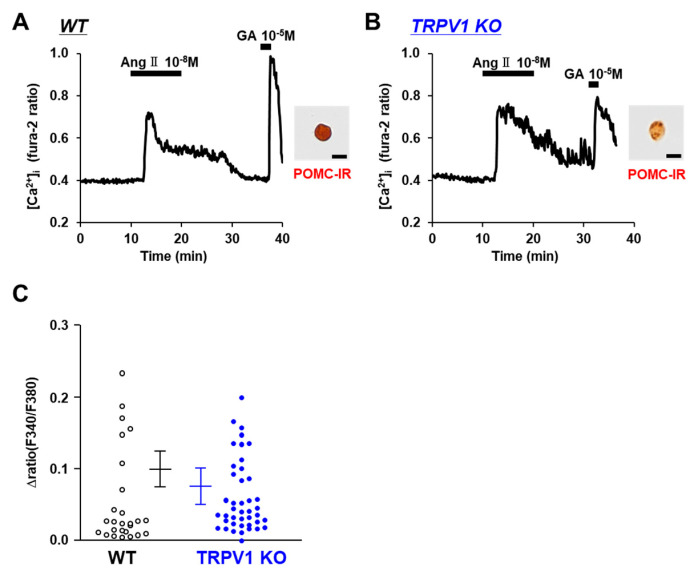
Angiotensin II increased [Ca^2+^]_i_ in single POMC neurons. (**A**,**B**) Angiotensin II increased [Ca^2+^]_i_ in single POMC neurons from wild-type (WT) mice (**A**) and TRPV1 KO mice (**B**). Scale bar is 20 μm. (**C**) Average amplitude of [Ca^2+^]_i_ responses to angiotensin II (Δratio) in POMC neurons. Data are presented as mean ± SEM. WT vs. KO determined by one-way ANOVA followed by the Bonferroni test.

The systemic renin–angiotensin system regulates fluid balance and blood pressure. Angiotensin II receptors, AT1R and AT2R, are not only located in the heart, lung, kidney, and blood vessel but also in the brain. Angiotensin II functions through both AT1R and AT2R in the brain to control fluid homeostasis and the autonomic pathways regulating the cardiovascular system. The neurons monitoring osmolality in the subfornical organ (SFO) are activated not only by the elevation in the extracellular [Na^+^] but also by angiotensin II [29]. We explored the effect of angiotensin II on POMC neurons. Angiotensin II at 10^−8^ M increased the [Ca^2+^]_i_ in the POMC neurons of both the C57BL/6 mice (Figure 6A) and TRPV1 KO mice (Figure 6B). The amplitude of the [Ca^2+^]_i_ increases in the POMC neurons were not significantly different between the C57BL/6 and TRPV1 KO mice (Figure 6C). These results suggest that angiotensin II increases [Ca^2+^]_i_ in POMC neurons via mechanisms that are independent of TRPV1. 

### 3.6. High Salt Intake Decreases POMC mRNA Expression in TRPV1 KO Mice

**Figure 7:**
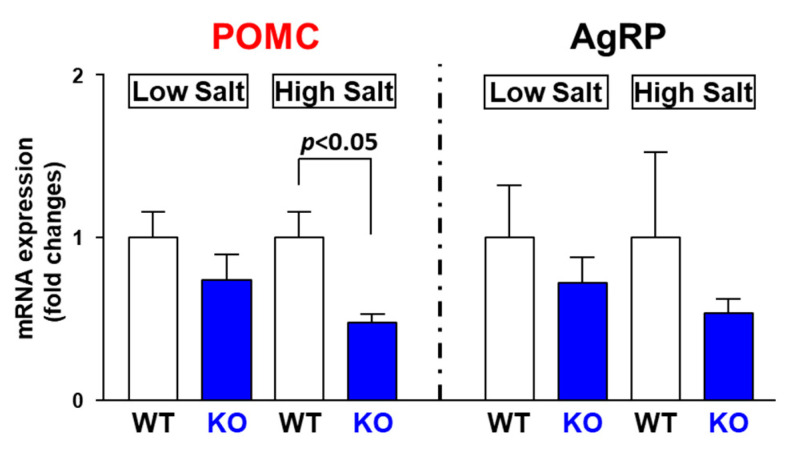
Relative mRNA expression (fold changes) in AgRP and POMC in ARC after 24 h of being fed a high-salt diet. WT mice and TRPV1 KO mice were fed either a low-salt diet (0.3% NaCl) or high-salt diet (8% NaCl) for 24 h. The expression of POMC in ARC was significantly decreased after 24 h of a high-salt diet in TRPV1 KO mice. □, WT mice; ■, TRPV1 KO mice. N = 6 for each group. Data are presented as mean ± SEM. WT vs. KO determined by one-way ANOVA followed by the Bonferroni test.

The effect of excessive salt intake on mRNA expression in the POMC and AgRP neurons in the ARC was examined using mice fed either a normal or high-salt diet for 24 h. In WT mice, excessive salt intake did not affect AgRP and POMC expression (Figure 7). However, in the TRPV1 KO mice, the mRNA expression of POMC but not AgRP was significantly decreased by the high-salt diet. These results indicate that TRPV1 could be implicated in maintenance of POMC neuronal activity in high-salt feeding conditions.

## 4. Discussion

The present study demonstrated that increased extracellular [Na^+^] activated the ARC POMC neurons of wild-type mice and that the increased [Na^+^]-induced activation of POMC neurons was abolished in TRPV1 KO mice. The ARC POMC neurons also responded to metabolic sugar, glucose, non-metabolized sugar, and mannitol. The ARC POMC neurons from the TRPV1 KO mice responded to the metabolic sugar but not the non-metabolized sugar. These results show that the ARC POMC neurons are capable of sensing extracellular Na^+^ and osmotic pressure via mechanisms involving TRPV1. Furthermore, the POMC neurons also sensed an osmoregulatory peptide, Angiotensin II. It is speculated that a subpopulation of ARC POMC neurons has multifaceted capabilities for sensing and regulating body fluid osmolarity.

It has been shown that high-salt food increases [Na^+^] in CSF as well as in plasma. The [Na^+^] concentration increased from 145 mM to 160 mM in the CSF of mice fed a high-salt diet [15]. Furthermore, an acute and physiological change in the plasma or CSF sodium concentrations (5–10 mmol/L) can activate the SNA and raise arterial blood pressure [16]. However, it is uncertain whether the increase in [Na^+^] is sensed by POMC neurons directly, even though these neurons express several receptors, transporters, and ion channels that are implicated in sensing circulating metabolic substrates, including glucose, amino acids, and fatty acids [4]. It is well known that the subfornical organ (SFO) and the organum vasculosum of the lamina terminalis (OVLT) of the sensory circumventricular organs (sCVOs) lack the blood–brain barrier and that the neurons in these areas can continuously monitor the [Na^+^] levels in body fluids [30]. The blood–brain barrier (BBB) is also immature in the ARC [31]. Notably, it has recently been reported that some neurons in the ARC are not insulated by the BBB and can be directly exposed to peripheral metabolic signals via a pathway involving tanycytes [32,33]. These reports suggest that the ARC neurons may have easy access to [Na^+^] changes in the blood. The present study first demonstrates that an increase in the extracellular [Na^+^] by 10 mM activates the ARC POMC neurons. This result shows the capability of the ARC POMC neurons to directly sense [Na^+^] in blood. 

It was reported that the TRPV1 in POMC neurons serves to maintain the ability to respond to changes in body temperature [22]. However, other possible functions of TRPV1 in POMC neurons remain unknown. The present results show a new role of TRPV1 in sensing extracellular [Na^+^] in POMC neurons. TRPV1 and TRPV4 can assemble into heteromeric complexes in the neurons in PVN and act as a sensor for warm temperatures [34]. TRPV4, in collaboration with the voltage-gated Na^+^ channel SCN7A, plays an important role in Na^+^ sensing by the neurons in the SFO and OVLT. Subpopulation of POMC neurons also express SCN7A and TRPV4 [21]. In these POMC neurons, TRPV1 could modulate the SCN7A/TRPV4 pathway, serving to monitor extracellular [Na^+^].

Under high-salt feeding conditions, the expression of POMC was reduced in the ARC of TRPV1 KO mice compared to wild-type mice. There are two possible causes for this result. First, the increases in [Na^+^] were incapable of activating the POMC neurons in the TRPV1 KO mice. Second, excessive salt intake activated the AgRP neurons but not the POMC neurons from the TRPV1 KO mice. AgRP neurons release the inhibitory neurotransmitters GABA and neuropeptide Y (NPY) and suppress the neurons to which they project, including ARC POMC neurons [35,36,37]. In the present study, we examined the effects of short-term (24-h) high salt diet loading, but longer-term loading should be further investigated.

The renin–angiotensin system (RAS) is thought to provide a link between the peripheral hormonal states and the central neurocircuits that control blood pressure. In the present study, angiotensin II activated the POMC neurons of wild and TRPV1 KO mice. Angiotensin II does not cross the BBB but is able to access the ARC due to the fragile property of the BBB there [38] and possibly via tanycyte-mediated transport [32]. The BBB permeability of angiotensin II in the ARC is increased in spontaneously hypertensive rats [39]. Furthermore, the action of angiotensin II in the ARC has been implicated in the regulation of neuroinflammation associated with obesity [40]. One angiotensin II receptor, AT2R, is specifically present in glutamatergic POMC neurons in the ARC [41], but its role is uncertain. Future studies on the function of AT2R in POMC neurons could provide a new approach to improve both metabolic and cardiovascular functions in obesity.

In this study, increased extracellular [Na^+^] also activated the AgRP neurons of the ARC. The AgRP neurons not only regulate energy metabolism but also cardiovascular functions [1,3,7]. Studies using designer receptors activated by designer drug (DREADD) technology have indicated that the activation of AgRP neurons tonically increases SNA and lowers blood pressure [42]. We previously reported that the deletion of phosphoinositide-dependent kinase-1 (PDK1) in AgRP neurons enhanced SNA, thereby exacerbating hypertension under high-salt feeding conditions [43]. In the present study, a fraction of the AgRP neurons responded to both excessive Na^+^ and the administration of nonmetabolized sugar, i.e., hyperosmotic conditions. This subpopulation of AgRP neurons may play a role in sensing the changes in the fluid osmolarity and/or [Na^+^] and thereby in regulating SNA and cardiovascular functions. Further elucidation of the mechanisms by which AgRP neurons sense the changes in fluid osmolarity and/or [Na^+^] could contribute to the prevention and treatment of hypertension and cardiovascular diseases.

## Data Availability

All data are available in this paper.

## Supplementary Materials

The following supporting information can be downloaded at: https://www.mdpi.com/article/10.3390/nu14132600/s1, Figure S1: Population of neurons in ARC of wild type mice was imaged.

## Abbreviations

ARC: arcuate nucleus of the hypothalamus; AgRP, agouti-related peptide; POMC, pro-opiomelanocortin; TRPV1, transient receptor potential vanilloid 1; KO, knockout.
