# Supplementary material for: TRPV1-Mediated Sensing of Sodium and Osmotic Pressure in POMC Neurons in the Arcuate Nucleus of the Hypothalamus
Source: Nutrients. 2022 Jun 23;14(13):2600. doi: 10.3390/nu14132600 (PMC9268643; doi:10.3390/nu14132600)
Supplement: Supplementary file 1 [file nutrients-14-02600-s001.zip › nutrients-1750439-supplementary.pdf]

A B

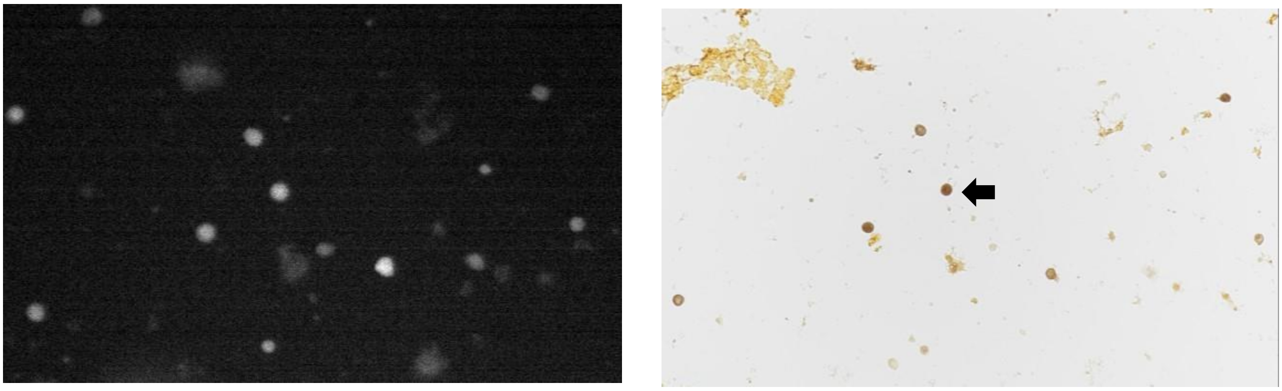

Supplemental Figure S1.

Population of neurons in ARC of wild type mice was imaged. A, a Fura-2 fluorescent image measured at 380-nm after $[Ca^{2+}]_i$ measurement. B, immunocytochemistry of AgRP. The neuron pointed by the arrow is the neuron in Figure 1A.
